# Amplicon-Based Next Generation Sequencing for Rapid Identification of *Rickettsia* and Ectoparasite Species from Entomological Surveillance in Thailand

**DOI:** 10.3390/pathogens10020215

**Published:** 2021-02-16

**Authors:** Suwanna Chaorattanakawee, Achareeya Korkusol, Bousaraporn Tippayachai, Sommai Promsathaporn, Betty K. Poole-Smith, Ratree Takhampunya

**Affiliations:** 1 Department of Entomology, US Army Medical Directorate of the Armed Forces Research Institute of Medical Sciences (USAMD-AFRIMS), Bangkok 10400, Thailand; suwanna.cho@mahidol.ac.th (S.C.); achareeya.korkusol@gmail.com (A.K.); BousarapornT.ca@afrims.org (B.T.); SommaiP.ca@afrims.org (S.P.); Betty.Poolesmith.mil@afrims.org (B.K.P.-S.); 2 Department of Parasitology and Entomology, Faculty of Public Health, Mahidol University, Ratchawithi Road, Bangkok 10400, Thailand

**Keywords:** Rickettsia asembonensis, Ctenocephalides felis orientis, Next-generation sequencing (NGS)

## Abstract

Background: Next generation sequencing (NGS) technology has been used for a wide range of epidemiological and surveillance studies. Here, we used amplicon-based NGS to species identify *Rickettsia* and their arthropod hosts from entomological surveillance. Methods: During 2015–2016, we screened 1825 samples of rodents and ectoparasites collected from rodents and domestic mammals (dog, cat, and cattle) across Thailand for *Rickettsia*. The citrate synthase gene was amplified to identify *Rickettsia* to species, while the Cytochrome Oxidase subunit I (*CO*I) and subunit II (*CO*II) genes were used as target genes for ectoparasite identification. All target gene amplicons were pooled for library preparation and sequenced with Illumina MiSeq platform. Result: The highest percentage of *Rickettsia* DNA was observed in fleas collected from domestic animals (56%) predominantly dogs. Only a few samples of ticks from domestic animals, rodent fleas, and rodent tissue were positive for *Rickettisia* DNA. NGS based characterization of *Rickettsia* by host identified *Rickettsia asembonensis* as the most common bacteria in positive fleas collected from dogs (83.2%) while “*Candidatus* Rickettsia senegalensis” was detected in only 16.8% of *Rickettsia* positive dog fleas. Sequence analysis of *CO*I and *CO*II revealed that almost all fleas collected from dogs were *Ctenocephalides felis orientis*. Other *Rickettsia* species were detected by NGS including *Rickettsia heilongjiangensis* from two *Haemaphysalis hystricis* ticks, and *Rickettsia typhi* in two rodent tissue samples. Conclusion: This study demonstrates the utility of NGS for high-throughput sequencing in the species characterization/identification of bacteria and ectoparasite for entomological surveillance of rickettsiae. A high percentage of *C. f. orientis* are positive for *R. asembonensis*. In addition, our findings indicate there is a risk of tick-borne Spotted Fever Group rickettsiosis, and flea-borne murine typhus transmission in Tak and Phangnga provinces of Thailand.

## 1. Introduction

The genus *Rickettsia* is comprised of obligate intracellular Gram-negative organisms that cause diseases in people throughout the world. Rickettsioses are zoonotic diseases that can be transmitted to human by ectoparasites including fleas, ticks, mites, and lice. Mammals including rodents, domestic animals, and wildlife species have been recognized as reservoirs of rickettsioses. Currently there are 30 identified *Rickettsia* species [1], and some of these species have been recognized as human pathogens [2]. *Rickettsia* are classically characterized into two groups, the spotted fever group (i.e., *Rickettsia rickettsii*, *Rickettsia africae*) which cause the tick-borne spotted fever group (SFG) rickettsioses, and typhus group (*Rickettsia prowazekii*, and *Rickettsia typhi*) which cause louse-borne epidemic typhus, and flea-borne murine or endemic typhus, respectively. There are also some pathogenic rickettsial species classified into the transitional group, as they share features between SFG and typhus group. The transitional rickettsiae includes *Rickettsia akari, Rickettsia australis, Rickettsia felis* which are causative agents of mite-borne rickettsialpox, Queensland tick typhus, and flea-borne spotted fever, respectively [2].

The epidemiology of *Rickettsia* spp. worldwide is defined by a combination of climate, the geographical distribution of the primary vector and distribution of the natural animal hosts required to amplify and maintain its presence in the environment. Most SFG *Rickettsia* are distributed in the limited geographic regions where their specific tick host species is found [2,3,4]. For example, Rocky Mountain spotted fever (RMSF) is caused by *R. rickettsii*, is distributed throughout the Americas, and *Dermacentor* ticks serve as the primary vector for RMSF in southeast and south central United States [5], while *Amblyomma cajennense* sensu lato species complex transmit RMSF in central and South America [6]. However, *Rhipicephalus sanguineus* sensu lato, which commonly infests dogs, has been implicated in an RMSF outbreak in the tribal lands of Arizona and in Sonora, Mexico [7,8]. Mediterranean spotted fever, caused by *Rickettsia conorii*, is distributed across southern Europe, northern Africa, with occasional cases reported in Asia, with the potential vector *R. sanguineus* s.l. (brown dog tick) [5,9]. *Rickettsia africae* causes African tick bite fever in sub-Saharan Africa which correlates with the distribution of its known vectors *Amblyomma hebraeum* and *Amblyomma variegatum* [10].

Although, there are a number of published reports of *Rickettsia* in Thailand, SFG *Rickettsia* are considered to be an under recognized cause of febrile illness in Thailand [11] and broader multi-province surveillance for *Rickettsia* vectors and animal reservoirs to better understand transmission and epidemiology is lacking. Briefly, there is serologic and molecular evidence that *Rickettsia honei*, and *Rickettsia japonica* cause humans infections in Thailand, [12,13,14]. In addition *R. honei* has been identified from *Ixodes* spp. and *Rhipicephalus* spp. larval ticks, and detection of *R. japonica* from *Haemaphysalis hystricis* suggesting these tick species as potential vectors [15,16]. Flea-borne murine typhus caused by *R. typhi,* and flea-borne SFG disease caused by *R. felis* are distributed worldwide. Murine typhus is endemic in Thailand, as shown to be the second leading cause of acute undifferentiated febrile illness in Bangkok [17]. *Rickettsia typhi* is transmitted by rat flea (*Xenopsylla cheopis*), and *Rattus* spp. serve as the primary reservoir [18]. In some regions, *R. typhi* can also be transmitted by cat flea (*Ctenocephalides felis*) [19,20]. The detection of *R. felis* and *R. felis*-like *Rickettsia* spp. DNA in dog, flea, and febrile patients suggests these bacteria can infect in human and domestic animals in Thailand [21,22,23].

To better understand the epidemiology of rickettsiosis and assess the risk for emerging rickettsial zoonoses in human, we performed surveillance for *Rickettsia* in wildlife and domestic animals, as well as their ectoparasites. Most studies perform PCR and sequence analysis of target genes to screen and characterize *Rickettsia* species and other interested pathogens [21,22,23,24,25]. While other studies applied a metagenomic approach using universal 16S rRNA sequencing to characterize a large spectrum of bacterial pathogens and bacterial communities in vectors and wildlife [26,27,28,29]. Due to high number of samples collected from surveys, high-throughput sequencing would have an advantage over the conventional Sanger sequencing for bacteria species characterization in large number of positive samples. Moreover, it would allow for molecular identification of ectoparasites and host species in parallel. In our previous surveillance during 2015–2016 in Thailand, a high percentage of flea samples were positive for *Rickettsia* DNA by real-time PCR assay. Here, we develop the amplicon-based next generation sequencing (NGS) to characterize *Rickettsia* and their arthropod hosts into species. Amplicons of the target genes for *Rickettsia* and ectoparasites species identification were sequenced simultaneously using Miseq platform. This alternative approach facilitates identification of *Rickettsia* spp. from a large number of positive samples acquired from our multi-province surveillance. The identification of bacteria, vector, and animal reservoirs would aid our understanding of the risk of rickettsial disease transmission and epidemiology.

## 2. Results

### 2.1. A High Percentage of Rickettsia DNA in Fleas Collected from Domestic Dogs

One hundred and three samples were PCR-positive for *Rickettsia* DNA from a total of 1825 samples including rodents, and ectoparasites collected from rodents and domestic animals, dog, cat, and cattle, (2015–2016). Table 1 shows the number of samples and the percentage of *Rickettsia* DNA by sample type and province where samples were collected. There was a high percentage of *Rickettsia* DNA in fleas were collected from domestic animals (97/173 = 56%), all positive fleas were collected from dogs. Of other sample types, only a few tick pools from domestic animals (3/157 = 1.9%), rodent flea pools (1/52 = 1.9%), and rodent tissue samples (2/1384 = 0.1%) were *Rickettsia* positive. No *Rickettsia* were found in lice collected from domestic animals or ticks collected from rodents. The data of domestic animals infested with ectoparasites were provided as Supplementary Materials (Tables S1 and S2).

### 2.2. Rickettsia Species in Rodent and Ectoparasite Pools

**Figure 1:**
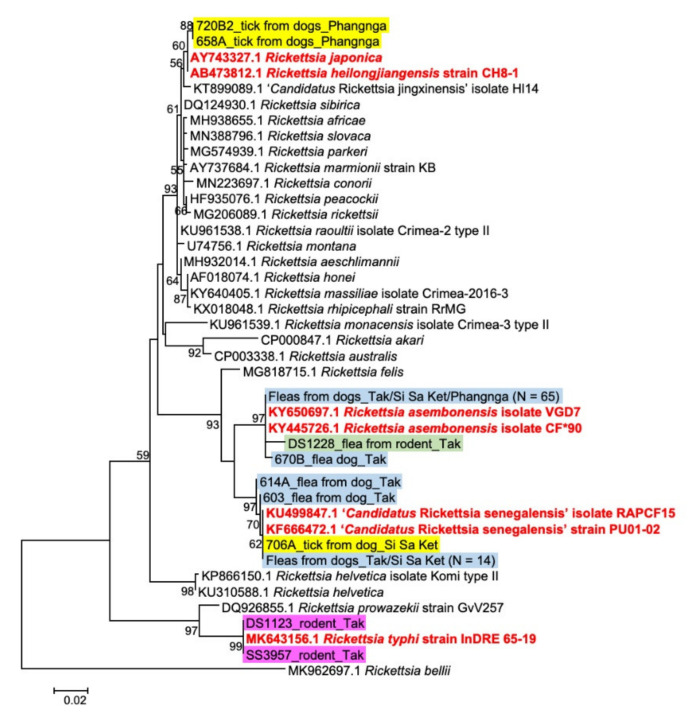
Phylogenetic tree of *Rickettsia glt*A gene detected in rodents and ectoparasites were analyzed with reference sequences retrieved from GenBank database. A maximum likelihood (ML) tree was constructed using the T92 + G models of nucleotide substitution in the MEGA 6 program with bootstrapping (1000 replicates). *Rickettsia* sequences from rodent, rodent fleas, flea, and ticks from dogs are highlighted in pink, green, blue, and yellow, respectively, and the provinces samples collected are indicated. The *Rickettsia* reference sequences of species identified in the present study are marked in red.

From a total of 103 *Rickettsia*-positive samples, 82 samples were selected for amplicon-based NGS species identification for *Rickettsia* species and Sanger sequencing was used for the remaining pools. *Rickettsia glt*A sequences were obtained from 101 samples. Phylogenetic analysis of *glt*A sequences for *Rickettsia* species characterization is shown in Figure 1. The *Rickettsia* species were identified based on their highest DNA sequence identity to the reference sequences retrieved from Genbank database. There were four *Rickettsia* species identified from our samples, *R. asembonensis*, “*Candidatus* Rickettsia senegalensis”, *R. helongjiangensis* and *R. typhi* with 99.2–100% identity to references sequences. The result of *Rickettsia* species characterization by *glt*A gene is shown in Table 2. The majority of *Rickettsia* species found were identified as *R. asembonensis* (80/101, 79.2%) followed by “*Ca.* R. senegalensis” (16.8%), *R. helongjiangensis* (2%) and *R. typhi* (2%). All *Rickettsia* from rodents (*n* = 2) and rodent fleas (*n* = 1) were *R. typhi* and *R. asembonensis*, respectively. The majority of *Rickettsia* identified from fleas collected from dogs (*n* = 95) were *R. asembonensis* (79/95, 83.2%), while “*Ca.* R. senegalensis” was detected in 16.8% (16/95) of fleas from dogs. Of three *Rickettsia*-positive ticks collected from dogs, two from Phangnga province were *R. helongjiangensis* and one from Si Sa Ket province was “*Ca.* R. senegalensis”.

### 2.3. Ectoparasite Species Identification in Rickettsia-Positive Pools

**Figure 2:**
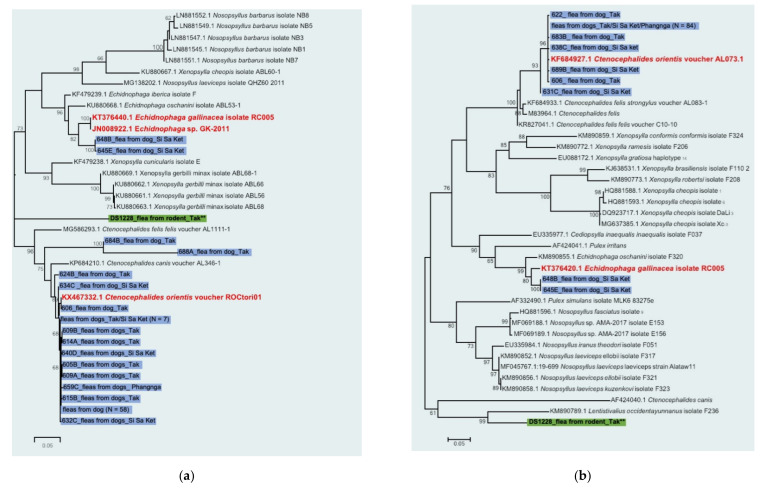
Phylogenetic trees of Cytochrome Oxidase subunit I (**a**) and II (**b**) sequences of *Rickettsia* positive fleas and reference sequences retrieved from GenBank database. A ML tree was constructed using the GTR + G models of nucleotide substitution in the MEGA 6 program with bootstrapping (1000 replicates). Sequences from fleas collected from rodent and dogs are highlighted in green and blue, respectively, and the provinces samples collected are indicated. The reference sequences of flea species identified in the present study are marked in red. Sequence classified as unknown species is noted**.

Results of flea species identification using *CO*I and *CO*II sequences by NGS and Sanger sequencing were available for 92 *Rickettsia*-positive pools (91 flea pools from dogs and 1 flea pool from rodent), and phylogenetic analysis was shown in Figure 2a,b respectively. The following results were obtained from *CO*I and *CO*II characterization for almost all flea samples (Table 3). The majority of *Rickettsia*-positive fleas from dogs (89/91 = 97.8%) were identified as *Ctenocephalides felis orientis* (*C. f. orientis*) with 98–100% identity to the reference sequence, only 2 of them (2.2%) were *Echidnophaga gallinacea.* (95–97% identity). Interestingly, two flea pools from dogs were unidentified in which their *CO*I sequences have only 91 and 82% identity to *C. f. orientis* sequence reference, but their *CO*II sequences are 100% identical to *C. f. orientis*. Thus, they are classified as *C. f. orientis*. One positive rat flea was classified as an unidentified group by both *CO*I and *CO*II gene sequences.

For tick species identification, only *CO*I sequence was used to characterize three *Rickettsia*-positive tick pools by Sanger sequencing. Two tick pools were characterized as *H. hystricis* with 99% identity to the reference sequence (data not shown), while one tick pool that had been previously identified as *R. sanguineus* s.l. by morphology failed in sequencing. When the distribution of *Rickettsia* species among ectoparasite species was examined, *R. asembonensis* and “*Ca.* R. senegalensis” were found in various species of fleas and *Rhipicephalus* ticks from dogs. Among *C. f. orientis* population (*n* = 89), 83% (74/89) carried *R. asembonensis* and 17% (15/89) had “*Ca.* R. senegalensis”. However, it was noted that *R. heilongjiangensis* was found in only *H. hystricis* ticks.

## 3. Discussion

Here, we demonstrated the utility of next generation sequencing (NGS) for high-throughput sequencing to identify *Rickettsia* and ectoparasite species in parallel. In our previous surveillance during 2015–2016 in Thailand, high percentage of flea samples were PCR-positive for *Rickettsia* DNA. Instead of using Sanger sequencing to characterize *Rickettsia* and flea species using their specific target genes which would require large amounts of time to assemble and analyze each individual sequence, we applied the amplicon-based NGS technology. The Citrate synthase (*glt*A) gene, Cytochrome Oxidase subunit I (*CO*I) and subunit II (*CO*II) amplicons were sequenced simultaneously using Miseq platform. The advantage of NGS over traditional Sanger sequencing is that amplicons of several target genes could be sequenced simultaneously and up to 384 samples could be analyzed in one experimental run, if dual indexes were used in library preparation (Illumina). NGS sequencing strategies are used widely and provide a range of applications for epidemiological and surveillance analysis. For example, whole genome sequencing and genome-wide screening for single nucleotide polymorphisms have been used for microbial typing for outbreak analysis [30]. Other researchers have used a metagenomic approach based on universal 16S rRNA sequencing to survey a large spectrum of bacterial pathogens and analyze bacterial communities in human, wildlife, and vectors [26,27,28,29]. The results of metagenomic approaches help elucidate the complexities of transmission and circulation of bacteria in the environment. Our study illustrates another application of NGS for simultaneous characterization of *Rickettsia* and flea species, to enable rapid analysis of a large number of samples collected from a surveillance study.

More than a half of all fleas from dogs during our 2015–2016 surveillance were PCR-positive for *Rickettsia* species and most were identified later as *R. asembonensis*. Molecular identification of flea species indicates all flea from dogs are *Ctenocephalides felis orientis*, except for two *Echidnophaga gallinacea* (sticktight flea) that may occasionally be found in dogs [31]. The evidence of high percentage of *C. f. orientis* positive for *R. asembonensis* correspond to the study in India, suggesting a specific host-bacteria relationship [32,33]. Similarly, the high percentage of *R. asembonensis* DNA were also detected among fleas from cats in Khon Kaen province, northeastern Thailand [23], although all fleas were identified as *C. felis* by morphology rather than molecular identification. *Rickettsia asembonensis* belongs to a group of *R. felis*-like organisms which are ubiquitously distributes. It was firstly detected as *Rickettsia* sp. RF2125 in *Ctenocephalides canis* in western Thailand near the Myanmar border [34]. Its host includes various arthropods but mostly in fleas of three families: *Pulicidae, Ceratophyllidae,* and *Coptopsyllidae* [33]. *Rickettsia asembonensis* could be a potential human pathogen as it has been reported from patients with fever of unknown origin in Thailand, Malaysia, and Peru [22,35,36,37]. *Rickettsia asembonensis* was also detected in healthy dogs in South Africa [38], sick dog in Thailand [22], and health monkey in Malaysia [39]. Nonetheless, its pathogenicity to humans and animals remains unknown.

A low percentage of *Rickettsia* DNA (<2%) was observed in dog tick, rodent flea, and rodent samples. Interestingly, *R. heilongjiangensis* was found in only *H. hystricis* ticks from dog in Phangnga province, while *R. typhi* was found in rodent samples from Tak province. *R. heilongjiangensis* belonging to Spotted Fever group (SFG), cause Far-Eastern spotted fever which is endemic in northeastern China [40], Siberia, and far-eastern Russia [41]. In Thailand, there has been one case report of SFG rickettsiosis in human related to *R. japonica*, later confirmed to be *R. heilongjiangensis* [14,16]. However, the identification of this agent in tick vector has not been documented. To our knowledge, this is the first report identifying *R. heilongjiangensis*, causative agent of SFG rickettsiosesis, in *H. hystricis* ticks in Thailand. Spotted Fever Group *Rickettsia* apparently adapts itself to a specific tick species, and the relationship between SFG *Rickettsia*, tick, and vertebrate host have been documented [5].

This study had several limitations: no access to blood from domestic animals and phylogenetic analysis was limited. The conclusions that can be drawn from the animal infections was limited as we were unable to collect blood samples from domestic cats and dogs. The phylogenetic analysis was limited to one or two genes with analyses of *Rickettsia* based on the Citrate synthase (*glt*A) gene (Table 1) and fleas on the Cytochrome Oxidase subunit I and II genes (Table 2). The *glt*A gene is best for distinguishing between *Rickettsia* species while the *CO*I gene is the gold standard for barcoding or distinguishing between species. This limits the breadth of the conclusions that can be drawn from these data to species identification. Therefore, we recommend that selection of genes be tailored to the study goal.

In conclusion, a high percentage of *R. asembonensis* DNA (>50%) was observed in fleas from dogs (*C. f. orientis*). *Rickettsia asembonensis* belongs to a group of *R. felis*-like organisms which distribute ubiquitously, but its definite pathogenicity to human remains unknown. We also identified *R. heilongjiangensis*, causative agent of SFG rickettsioses, in *H. hystricis* ticks from dog in Phangnga province and *R. typhi* in rodents from Tak province. Our findings demonstrate the circulation of tick- and flea-borne SFG rickettsioses, and flea-borne murine typhus, and thus risk of transmission from potential vectors and animal reservoir in Tak and Phangnga provinces of Thailand. Moreover, our study illustrates another application of NGS for simultaneous characterization of *Rickettsia* and flea species, to enable rapid analysis of a large number of samples collected from a surveillance study.

## 4. Materials and Methods

### 4.1. The Field Surveillance for Rickettsia in Rodents and Ectoparasites

During field surveillance for *Rickettsia* species (2015–2016), rodents and ectoparasites collected from rodents and domestic mammals (dog, cat, and cattle) in Phangnga, Ranong, Si Sa Ket, Tak, and Udon Thani provinces of Thailand were screened for *Rickettsia* spp. Rodents were captured from plantations, cultivated rice-fields, and grassland areas using live traps, as previously described [28]. Captured rodents were euthanized using carbon dioxide and processed immediately at the site of collection. Liver, spleen, kidney, and lung samples were collected and stored on dry ice. Ticks, fleas, and lice were collected and kept in absolute ethanol. Ectoparasites were also collected from domestic animals (dog, cat, and cattle). There were pools of ectoparasites (ticks, fleas, and lice) collected from individual rodent or domestic animal. All rodent and ectoparasite samples were transported to the AFRIMS laboratory, Bangkok, Thailand, for further processing. Rodent species were identified to species level as described previously [42]. Ectoparasites (ticks, fleas, and lice) were morphologically identified [43,44,45] and pooled by genus, stage, gender, and their animal host origin. Up to five ticks were included in a pool, while there were as many as 29 fleas and 11 lice from a domestic mammal included in a pool.

### 4.2. Genomic DNA Extraction from Rodents and Ectoparasite Samples

Genomic DNA was extracted from rodent tissue, and ectoparasite pools, as previously described [28]. Briefly, kidney and spleen tissue from each rodent were cut into small pieces, homogenized, and extracted for genomic DNA using a QIAsymphony^®^ SP instrument (Qiagen, Hombrechtikon, Switzerland) with QIAsymphony^®^ DNA Mini Kit (Qiagen, Germany). For ectoparasites, a modified protocol of QIAamp DNA Mini Kit (Qiagen) was used to extract genomic DNA from each pool as previously described [28]. DNA was kept in −20 °C until use.

### 4.3. Real-Time PCR Detection of Rickettsia

Rodent samples and ectoparasite pools were screened for *Rickettsia* using TaqMan real-time PCR (qPCR) targeting 17 kDa protein gene and some of the positive signals from qPCR were confirmed by conventional PCR of *Rickettsia* citrate synthase (*glt*A) gene. Details of primers, probes and PCR conditions were previously published by Takhampunya et al. 2019 [28].

### 4.4. Amplicon-Based NGS Characterization of Rickettsia and Ectoparasite Species

Most *Rickettsia*-positive ectoparasite pools were selected for NGS characterization of *Rickettsia* (82/103) and flea species (92/103), and the remaining pools was characterized by Sanger sequencing, while there was not enough DNA to do sequencing for two samples. Citrate synthase (*glt*A) gene was amplified for *Rickettsia* species characterization, while Cytochrome Oxidase subunit I (*CO*I) and subunit II (*CO*II) genes were used as target genes for flea species identification. The *glt*A amplification was done following the protocol for the *Rickettsia* confirmation assay as mention earlier [28]. Primer sequences for flea *CO*I and *CO*II and PCR conditions were described in the previous study [46,47]. The *glt*A, *CO*I, and *CO*II amplicons of each sample were pooled and subjected to NGS. Since the length of these amplicons is more than 600 bp, direct amplicon sequencing on Illumina system could not provide sequence information across the entire length. So, the Nextera XT DNA library prep kit was used to prepare amplicon libraries, according to the manufacturer’s protocol (Illumina). Briefly, amplicons were fragmented and tagged with adapter sequences by Nextera transposome. Then, library was amplified using a limit-cycle PCR to tag DNA fragments with dual indices and Illumina sequencing adapters. The final products were purified using Agencourt AMPure XP beads to remove excess primers, nucleotides, salts, and enzymes. The purity of the libraries was verified using the QIAxcel Advanced System (Qiagen) with a QIAxcel DNA High Resolution Cartridge. The purified Index library concentration was quantified using the Qubit dsDNA HS Assay Kit (Invitrogen) and DNA concentration was normalized to 4.0 nM for each library. Five microliters of DNA from each library at 4.0 nM were pooled for one NGS run. Pooled libraries were denatured with NaOH, according to the manufacturer’s protocol (Illumina). Denatured libraries were diluted to a final concentration of 4 pM. A 10% Phix control adapter-ligated library (Illumina) was included to serve as internal control of each run for low-diversity library. Sequencing was performed on the Illumina MiSeq System using the MiSeq Reagent Kit v2 (300-cycles), following manufacturer’s instructions. High throughput sequencing (HTS) datasets was deposited in the NCBI’s Sequence Read Archive under BioProject # PRJNA691717, accession number SRR13425361-SRR13425442.

For Sanger sequencing, the PCR product of each gene was sequenced. Excess primers and nucleotides were removed from PCR amplicons using ExoSAP-IT™ (Applied Biosystem) according to the manufacturer’s instructions. The purified amplicons were sequenced using an ABI BigDye™ Terminator v3.1 Cycle Sequencing Kit, ethanol precipitated, and run on a SeqStudio Genetic Analyzer (Applied Biosystems Thermo Fisher, Thailand). Sequences were assembled using Sequencher™ ver. 5.1 (Gene Codes Corp., Ann Arbor, MI, USA). Nucleic acid sequences generated during this study (Sanger sequences and consensus sequences from NGS) were deposited in the GenBank database (http://www.ncbi.nlm.nih.gov/Genbank/) under accession number MW492078-MW492178, MW492179-MW492261, and MW492262-MW492354. The full list of sequences and their database identifiers are given as Supplementary Materials (Table S3).

### 4.5. Sequence Analysis

The sequence reads generated on MiSeq sequencers were processed on the CLC Genomics workbench software v 20.0.3 (Qiagen, Aarhus A/S1). High-throughput sequences were imported into CLC Genomics Workbench according to quality scores of Illumina pipeline 1.8 (using a Phred scale encoded using ASCII 33 to 93). The sequences were trimmed to remove adaptor sequences, low quality sequence (limit = 0.05), ambiguous nucleotides (maximal 2 nucleotides allowed), and read lengths shorter than 100 bp. Trimmed reads were mapped to reference sequences of *Rickettsia glt*A, flea *CO*I, and *CO*II retrieved from Genbank (KY650697.1, KY865419.1, and M83964.1, respectively). Consensus sequences were extracted from read mapping, according to default settings, and used for phylogenetic tree analysis. Sequences from NGS and Sanger sequencing were aligned with reference sequences retrieved from the GenBank database using the MUSCLE codon alignment algorithm [48]. Maximum likelihood phylogenetic tree was then constructed using the best fit model of nucleotide substitution with bootstrapping (1000 replicates) in MEGA 6 [49].

## Figures and Tables

**Table 1:** A high percentage of *Rickettsia* DNA in rodent and ectoparasite pools collected in field surveillance during 2015–2016.

Sample Type/Provinces	*Rickettsia*-PCR Positive Pools (% Positive)					
	Phangnga 8°41′08.5″ N 98°21′07.8″ E	Ranong 10°21′54.7″ N 98°49′01.8″ E	Si Sa Ket 14°34′13.9″ N 104°20′25.9″ E	Tak 16°23′41.4″ N 98°44′03.7″ E	Udon Thani 17°36′44.3″ N 102°20′38.3″ E	Total
Rodent	0/516 (0%)	0/137 (0%)	0/220 (0%)	1/452* (0.2%)	1/59* (1.7%)	2/1384 (0.1%)
Rodent Fleas	0/3 (0%)	NA.	0/15 (0%)	1/22 (4.5%)	0/12 (0%)	1/52 (1.9%)
Rodent Tick	0/8 (0%)	NA.	0/12 (0%)	0/17 (0%)	NA.	0/37 (0%)
Rodent Lice	0/2 (0%)	NA.	0/1 (0%)	0/3 (0%)	NA.	0/6 (0%)
DA Flea^1^	16/21 (76.2%)	NA.	31/57 (54.4%)	50/95 (52.6%)	NA.	97/173 (56.1)
DA Tick^2^	2/88 (2%)	NA.	1/69 (1.4%)	NA.	NA.	3/157 (1.9%)
DA Lice^3^	0/5 (0%)	NA.	0/6 (0%)	0/5 (0%)	NA.	0/16 (0%)
**Total**	**18/643 (2.8%)**	**0/137 (0%)**	**32/380 (8.4%)**	**52/594 (8.8%)**	**1/71 (1.4%)**	**103/1825 (5.6%)**

DA = domestic animals (dog, cat, and cattle), NA. = no samples collected; ^1^ Of a total 173 DA Flea pools, 20 and 153 pools were from cats, and dogs, respectively; ^2^ Of a total 157 DA Tick pools, 3, and 154 pools were from cattle and dogs, respectively; ^3^ All 16 DA Lice pools were from dogs; *No ectoparasites were collected from two *Rickettsia*-PCR positive rodents.

**Table 2:** *Rickettsia* species identification by *Rickettsia* citrate synthase (*glt*A) gene in rodent samples and ectoparasite pools.

*Rickettsia* Species Identified	Sample Type				Total Positive Samples (% of Overall Positive Samples)
	Rodent	Rodent Flea	DA Flea	DA Tick	
“*Ca.* R. senegalensis”	0	0	16	1	17 (16.8%)
*R. asembonensis*	0	1	79	0	80 (79.2%)
*R. helongjiangensis*	0	0	0	2	2 (2.0%)
*R. typhi*	2	0	0	0	2 (2.0%)
**Total**	**2**	**1**	**95**	**3**	**101 (100%)**

DA = domestic animals (dog).

**Table 3:** Ectoparasite species identification by Cytochrome Oxidase subunit I (*CO*I) and subunit II (*CO*II) genes in *Rickettsia*-positive pools.

Host	Ectoparasite Species	*Rickettsia* Species			Total
		“*Ca.* R. senegalensis”	*R. asembonensis*	*R. heilongjiangensis*	
**Rodent**	Flea- Unknown species	0	1	0	1
**Dog**	Flea- *Ctenocephalides f. orientis*	15	74	0	89
	Flea- *Echidnophaga gallinacea*	1	1	0	2
	Tick- Unknown *	1	0	0	1
	Tick- *Haemaphysalis hystricis*	0	0	2	2
**Total**	**All species**	**17**	**76**	**2**	**95**

Note, (*) tick was identified as *Rhipicephalus sanguineus* sensu lato by morphology, but sequencing failed.

## Data Availability

The data presented in this study are available in the materials and methods section as well as in the Supplementary Materials (Table S3). High throughput sequencing (HTS) datasets was deposited in the NCBI’s Sequence Read Archive under BioProject # PRJNA691717, accession number SRR13425361-SRR13425442.

## Supplementary Materials

The following are available online at https://www.mdpi.com/2076-0817/10/2/215/s1, Table S1: The number of animals infested with ectoparasites as well as the number (percentage) of animals infested with *Rickettsia*-PCR positive fleas or ticks, Table S2: The number of dogs infested with ectoparasites and screened for *Rickettsia* DNA as well as *Rickettsia* species detected from ectoparasites collected from them, Table S3: The GenBank (http://www.ncbi.nlm.nih.gov/Genbank/) accession numbers for the *Rickettsia glt*A gene, Cytochrome Oxidase subunit I and II genes of *Rickettsia*-positive fleas described in this paper.

## Disclaimer

Material has been reviewed by the Walter Reed Army Institute of Research. There is no objection to its presentation and/or publication. The opinions or assertions contained herein are the private views of the author, and are not to be construed as official, or as reflecting true views of the Department of the Army or the Department of Defense. Research was conducted under an approved animal use protocol in an AAALACi accredited facility in compliance with the Animal Welfare Act and other federal statutes and regulations relating to animals and experiments involving animals and adheres to principles stated in the Guide for the Care and Use of Laboratory Animals, NRC.
