# Supplementary material for: Amplicon-Based Next Generation Sequencing for Rapid Identification of Rickettsia and Ectoparasite Species from Entomological Surveillance in Thailand
Source: Pathogens. 2021 Feb 16;10(2):215. doi: 10.3390/pathogens10020215 (PMC7920428; doi:10.3390/pathogens10020215)
Supplement: Supplementary file 1 [file pathogens-10-00215-s001.zip › Supplementary Materials/Supplementary Materials_Table S1-S2.pdf]

Table S1. The number of animals infested with ectoparasites as well as the number (percentage) of animals infested with *Rickettsia*-PCR positive fleas or ticks.

**Type of animal host**	**N**	**Number of animals infested with ticks**	**Number of animals infested with *Rickettsia*-PCR positive ticks**	**Number of animals infested with fleas**	**Number of animals infested with *Rickettsia*-PCR positive fleas**
Dogs	116	64	3 (4.7%)	84	68 (81.0%)
Cats	7	0	0	7	1 (14.3%)
Cattle	2	2	0	0	0

Table S2. The number of dogs infested with ectoparasites and screened for *Rickettsia* DNA as well as *Rickettsia* species detected from ectoparasites collected from them.

**Ectoparasite types**	**Number of dogs infested with ectoparasites**	**Number of animals infested with *Rickettsia*-PCR positive ticks or fleas**	**Number of dogs infested with *Rickettsia* species-PCR positive fleas (N=68)**		
			R. asembonensis	***Ca.* R. senegalensis**	***R. asembonensis* and *Ca*. R. senegalensis**
Ticks only	32	2 (6.3%)*	0	0	0
Fleas only	52	42 (80.8%)	31	5	6
Ticks and fleas	32	27 (84.4%)[#]	24	1	1
**Total**	**116**	**71 (61.2%)**	**55**	**6**	**7**

*One was infected with *Ca*. R. senegalensis, another was infected with *R. heilongjiangensis*; [#]Only one tick pool was positive for *R. heilongjiangensis*.
